# Bone diagenesis and stratigraphic implications from Pleistocene karst systems

**DOI:** 10.1038/s41598-025-88968-4

**Published:** 2025-02-14

**Authors:** Héctor Del Valle, Alejandro B. Rodríguez-Navarro, Abel Moclán, Paula García-Medrano, Isabel Cáceres

**Affiliations:** 1 Institut Català de Paleoecologia Humana i Evolució Social (IPHES-CERCA), Zona Educacional 4, Campus Sescelades URV (Edifici W3), 43007 Tarragona, Spain; 2 Departament d’Història i Història de l’Art, Universitat Rovira i Virgili, Avinguda de Catalunya 35, 43002 Tarragona, Spain; 3 Departamento de Mineralogía y Petrología, Universidad de Granada, 18002 Granada, Spain; 4 Laboratoire PALEVOPRIM, Université de Poitiers and CNRS, Poitiers, France; 5 Institute of Evolution in Africa (IDEA), University of Alcalá de Henares, Covarrubias 36, 28010 Madrid, Spain; 6 UMR 7194 HNHP (MNHN-CNRS-UPVD), Département Homme et Environnement, Muséum National d’Histoire Naturelle, Paris, France; 7 Department Britain, Europe and Prehistory, British Museum, London, UK

**Keywords:** Infrared spectroscopy, X-ray diffraction, Apatite, Fossil bones, Machine learning, Archaeology, Mineralogy

## Abstract

Bone diagenesis is a complex process that modifies bone components in response to burial conditions. These modifications help to understand deposit formation and classify fossils by stratigraphy. The combined techniques of X-ray diffraction with Rietveld refinement and infrared spectroscopy were used to study the bone diagenetic processes along the complete stratigraphic sequence of Galería site (Sierra de Atapuerca, Spain). Eleven chemometric indices considering the different bone components (phosphates, carbonates, organic phase), together with the apatite unit cell parameters and cell volume were evaluated by 9 machine learning algorithms for bone diagenesis/stratigraphic classification. The results showed differences along the stratigraphic sequence due to changes in the apatite structure chemistry (i.e., F^−^ and OH^−^), producing a gradual shift of the unit cell volume (from 531.9 to 526.1 Å^3^) from GII to GIV associated with coupled dissolution–precipitation processes. Two diagenetic pathways are indicated: The lowest unit (GII) is characterized by leaching and carbonate loss in bone, suggesting an acidic and wet burial environment with the formation of authigenic phosphate minerals. The uppermost units (GIII-GIV) show bone apatite undergoing F^−^ and CO_3_ incorporation, suggesting a slightly alkaline and drier environment. These differences enabled the development of classification models to understand deposit formation dynamics and also recontextualize dissociated fossil bones.

## Introduction

Bone is a complex composite biomaterial that fulfils several mechanical, physiological, and chemical regulatory functions, such as mineral ion homeostasis or cell storage^1–4^. Bone is largely composed of an organic phase and a mineral phase. The mineral phase, made up of carbonated apatite nanocrystals^5^, can be described as Ca_10−X_[(PO_4_)_6−x_(CO_3_)_x_](OH)_2−x_ nH_2_O^6^, whose non-stoichiometric composition includes multiple ionic substitutions, such as CO_3_^2−^, Na^+^, F^−^, Zn^2+^, Sr^2+^, and Ba^2+^^6^. Apatite has hexagonal symmetry (space group P6_3_/m) and contains 4–8% carbonate, substituting PO_4_^3−^ groups (B-type) or OH^−^ groups (A-type) in the crystal lattice^7^. The organic phase of bone is mainly composed of type I collagen together with non-collagenous proteins, proteoglycans, osteocalcin, and glycoproteins^5,8^. Proteins are intimately associated with the mineral part of the bone, controlling mineral formation and many of its properties^5,8^.

The modification of bone remains over time begins with the death and deposition of an organism. From this moment, the remains are affected by various processes. These can be divided into two main phases within the framework of taphonomic studies. The first is biostratinomy, encompassing all processes that occur before the sedimentation and burial of the remains. The second is fossil diagenesis, which includes the physical and chemical processes that occur after the sedimentation and burial. All taphonomic processes are framed by the environment in which they develop, thus being linked to their taphosystem^9,10^. Bone diagenetic processes are influenced by their burial environment. However, it is important to consider that many biostratinomic modifications (e.g., bone heating, digestion, or weathering) may also affect diagenetic processes (e.g., dissolution-(re) precipitation)^11–13^, altering the initial conditions of the fossil-diagenetic phase even within the same burial context. Due to the complexity of these processes, it is necessary to study them using complementary techniques such as histological, mineralogical, structural, and chemical analyses to fully characterize and understand them. While the taphonomic processes in the Sierra de Atapuerca sites have been thoroughly investigated from a biostratinomic perspective^14–19^, their fossil-diagenetic aspects remain less explored (e.g., Del Valle et al.^13^).

**Fig. 1:**
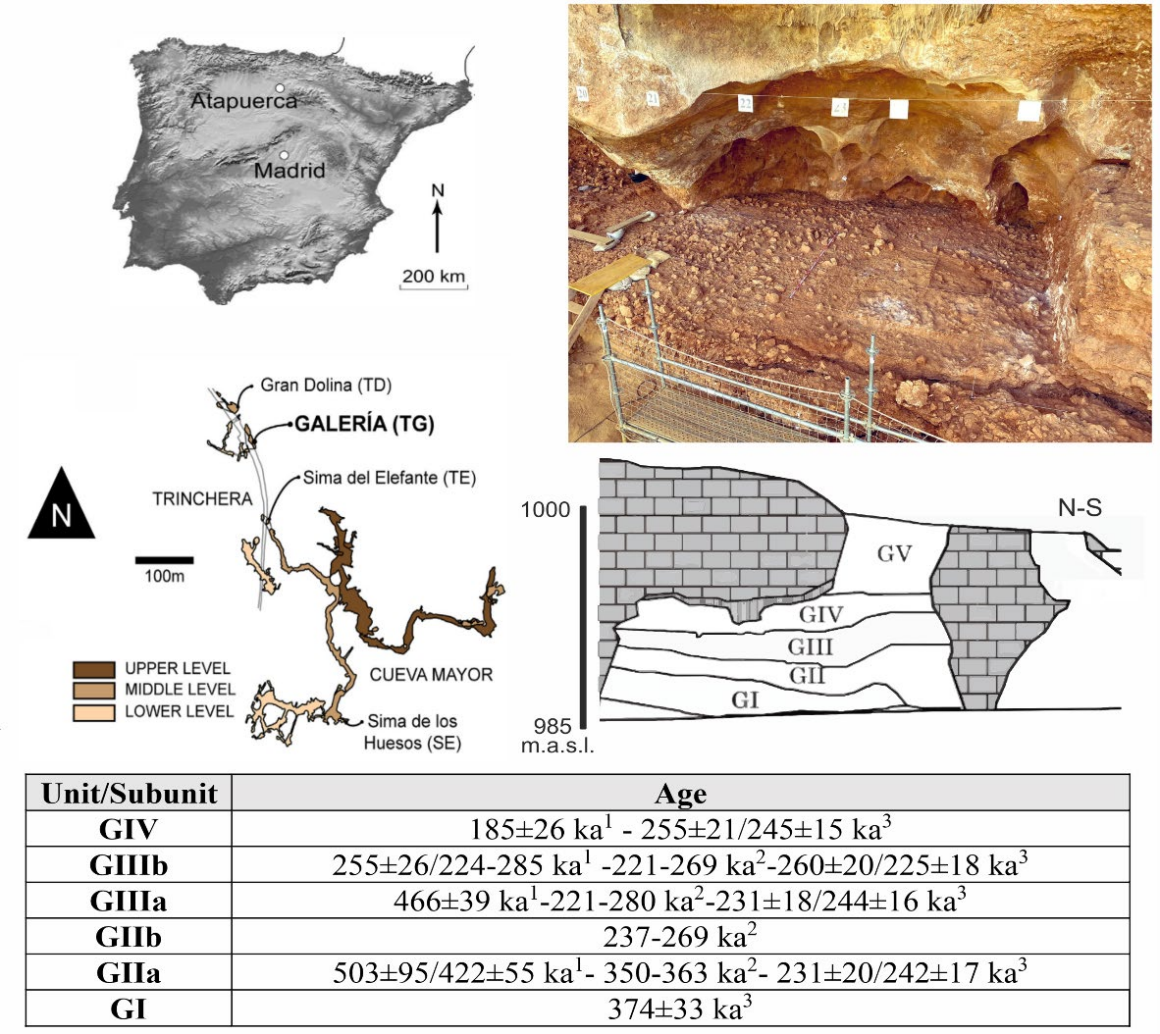
Location of the Galería site within the Las Torcas karst system (modified from^23,24^. Schematic karstic infill of the Galería site (modified from^25^ and age (1:^26^; 2:^27^; 3:^23^). Photograph of the current excavation surface on the top of GII (photograph by M. Guillén/IPHES-CERCA).

**Fig. 2:**
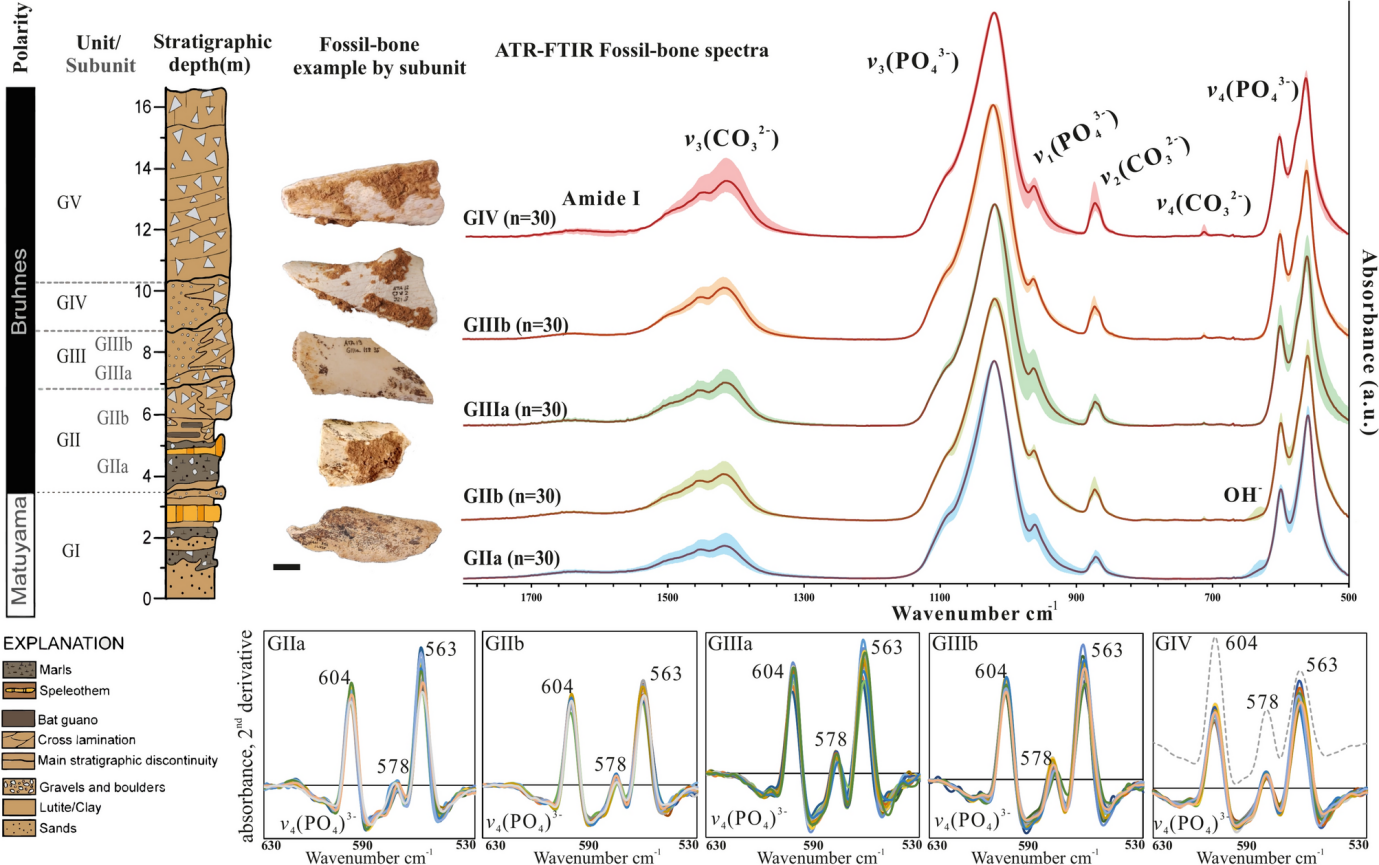
Average normalised ATR-FTIR spectra set for bone samples from each stratigraphic unit. Images of fossil bone examples of each unit and the stratigraphic column of the Galería site are shown alongside the spectra (modified from^24^). Below, detail on the second-derivative spectra of the region *v*_4_(PO_4_^3−^) for each unit/subunit. The grey dashed spectrum in the GIV chart shows an example of the same bands for fluorapatite.

In this study, we focus on the diagenetic processes studied from infrared spectroscopy (ATR-FTIR)^20,21^ and X-ray diffraction (XRD) based on the Galería site. Galería is one of the sites that makes up the archaeopalaeontological complex of the Sierra de Atapuerca (Burgos, Spain), which is located to the north of the northern plateau at an altitude of 1085 masl. It is composed of several cave systems. Specifically, Galeria, along with Gran Dolina and Sima del Elefante, is located on the western side of the Sierra within a railway trench (Fig. 1). It measures approximately 14 m high, 18 m wide, and over 12 m deep. The stratigraphic sequence belonging to the Middle Pleistocene is composed of six lithostratigraphic units named from GI to GV^22^ (Figs. 1 and 2). Detailed information on the site and each stratigraphic unit is provided in Supplementary Information 1 (SI 1).

The aims of this study are (1) to characterize bone diagenetic processes throughout a complete Pleistocene stratigraphic sequence using ATR-FTIR and XRD and (2) to determine whether differences in bone preservation exist among the stratigraphic units. This could be used to reconstruct the burial history and environmental conditions at the deposition site. Once the differences were understood, classification methods (3) were proposed to assign fossil bones to their corresponding geological units based on their chemical properties. This approach involves Principal Component Analysis (PCA) to evaluate the contribution of the variables, combined with supervised Machine Learning (ML) models for classification. These models are particularly significant as they provide a framework to recontextualize archaeological fossils which currently lack stratigraphic context.

## Material and methods

A total of 150 fossil bone samples have been analysed for this study (Table S1) from each Galeria unit and subunit (30 by unit/subunit) containing archaeopaleontological remains (GIIa, GIIb, GIIIa, GIIIb, and GIV). Each bone sample corresponds to cortical bone from anatomical elements of large and medium-sized fossil mammals (Table S1).

### Infrared spectroscopy and chemometrics

Fourier transform infrared spectroscopy (FTIR) is a powerful tool for understanding changes in the mineral and organic components at the molecular level. FTIR analysis was performed in attenuated total reflection (ATR) mode (Fig. 2). The advantages of using ATR-FTIR over the transmission mode for archaeological bones are evident as it is fast, requires minimal and fast preparation, a small amount of sample, and allows for the reduction of analytical errors^20,21^. In addition, it allows semi-quantitative chemometric analyses to be carried out between different bone remains or archaeological assemblages.

Each bone sample was processed following the methodology proposed by Kontopoulos et al.^28^ and Lebon et al.^29^. The spectra were collected on a Jasco FT/IR-6800 spectrometer with a resolution of 2 cm^−1^ and 64 scans in the range 4000–400 cm^−1^ at the SRCiT URV (University of Rovira i Virgili’s Scientific and Technical Service, Tarragona, Spain). Around 1 mg of sample powder was pressed on the surface of a diamond crystal with a 50–20 μm particle size^28^. The anvil pressure on the ATR crystal was adjusted to obtain a 0.5 absorbance for the ν_3_(PO_4_^3−^) band around 1010 cm^−1^^29^. Three spectra were analysed for each sample. Spectra analysis was performed using Spectra Manager™ 2.15.00 software (JASCO).

Overlapping peaks were resolved and their integrated areas were measured using specifically designed curve fitting software^30^. From the peak areas, the following compositional parameters were determined to define bone material properties: (1) *v*_1,_ *v*_3_(PO_4_^3−^) band area ratio (800–1200 cm^−1^).

For additional information, the second derivative was calculated using a Savitzky-Golay algorithm (2nd derivative order and 3 polynomial order) with 5 points of window for *v*_3_(PO_4_^3−^) and *v*_4_(PO_4_^3−^) spectral regions^31,32^.

After processing the spectra, the chemometric indices were obtained. The indices are shown in Table 1. These were chosen to describe the different taphonomic modifications in the main bone components (phosphates, carbonates, and organic phase).

More specifically, the indices that analyse phosphates’ crystallinity and behaviour in the spectrum include the infrared splitting factor (IRSF), which is the crystallinity index proposed by Weiner and Bar-Yosef^33^ and is calculated from the v_4_(PO_4_^3^⁻) band (IRSF = 600 cm⁻^1^ + 560 cm⁻^1^ / 590 cm⁻^1^). The highly crystalline phosphate index (HCP) and the poorly crystalline phosphate index (PCP), which are calculated from the area of the 1030 cm⁻^1^ and 960 cm⁻^1^ bands in the deconvoluted spectrum as presented by Rodríguez-Navarro et al.^34^, provide information on the degree of crystallinity and the order or disorder of phosphates in the crystal lattice. An increase in the intensity of the HCP indicates that the bone mineral has become more stable and structurally ordered, while a high PCP value reflects a less crystalline bone mineral. To understand the presence and behaviour of non-apatitic phosphates in different contexts, the band areas in the region between 1100 and 1200 cm⁻^1^, attributed to HPO_4_^2^⁻ in non-apatitic environments^34–36^, were used based on the deconvolution of the *v*_1_,*v*_3_(PO_4_^3^⁻) region. The FWHM of the *v*_3_(PO_4_^3^⁻) region around 1010 cm⁻^1^ was used to understand the order and strain in the crystal lattice, considering particle size and sample preparation^28^.

**Table 1:** Chemical parameters calculated from the analysis of bone through ATR-FTIR and used in this study.

Indices	Peaks (cm^−1^)	Description	Baseline correction (cm^−1^)	References
Amide I/PO_4_	1640 / 1010	Amide I to phosphate	1710–1590	^29,37^
			1150–890	
IRSF	(560 + 600) / 590	Infrared splitting factor. Indicate crystal size and order in the matrix	660/640 470/420	^33^
C/P	1410 / 1010	Carbonate to phosphate	1590—1290	^38^
C/C	1455 / 1410	A + B type carbonates band area in deconvoluted spectrum		^34,39^
FWHM(*ν*_3_PO_4_)	1010	Full width at half-maximun of the *ν*_3_(PO_4_) band, indicating lattice order	1150–890	^21,28^
OH/PO_4_	630 / 605	OH^−^ groups libration	660/640 470/420	^40^
Calcite/PO_4_	712 /1010	Calcite to phosphate	730–700	^41^
HPO_4_1118	Acid phosphate band area in deconvoluted spectra at 1118 cm^−1^			^34–36^
HPO_4_1145	Acid phosphate band area in deconvoluted spectra at 1145 cm^−1^			^35,36^
HCP	Highly crystalline phosphate, band area at 1030 cm^−1^ in deconvoluted spectra			^34^
PCP	Poorly crystalline phosphate, band area at 960 cm^−1^ in deconvoluted spectra			^34^

### X-ray diffraction (XRD)

The mineralogy and crystallinity of fossil bone were characterized by X-ray diffraction (XRD) using an Xpert Pro X-ray powder diffractometer (Panalytical) at the Universidad de Granada (Spain). Bone powder samples (approx. 0.2 g) were measured in reflection mode in a standard sample holder using copper radiation and a 5–120° 2Theta angular range (0.013° steps; 100 s integration time). Rietveld refinement analysis of XRD profiles was performed with TOPAS 5.0 software (Bruker). The Rietveld refinement was applied to calculate the apatite unit cell parameters and volume^31,42^. Mineral phases fitted were hydroxylapatite, calcite, and quartz. For hydroxylapatite, unit cell parameters were refined. Other relevant parameters (zero error) were also refined, and we got a goodness of fit (Rwp) between 5 and 7%.

### Statistical analysis

Statistical analyses were performed using R software version 4.2.2. The results were expressed as mean ± standard deviation (Table S2). The chemometric indices were first previsualised through violin plot diagrams and examined for normality using the Shapiro–Wilk test and Levene’s test to test homoscedasticity. Based on the results, the non-parametric Kruskal–Wallis test or Welch’s ANOVA test were used to compare the difference assemblage. After that, a Wilcoxon rank sum test for pairwise comparison was applied to discern the differences between assemblages. *P*-values ≤ 0.05 were considered statistically significant. Subsequently, a PCA, which is a linear combination of the original variables, was used to observe the distribution and importance of the variables. A PCA allows us to better understand which variables are more important in the distribution of the samples as well as allows us to better understand the trends of the diagenetic trajectories^43^.

Additionally, a correlation matrix was constructed to examine the associations between the variables. This is a valuable technique for managing multicollinearity in the dataset. If two or more variables exhibit a high correlation (values greater than 0.75 or − 0.75), it suggests that there is considerable redundancy in the information they provide for the classification models. For enhanced understanding of the data, the parameters for a modern adult pig’s cortical femur (*Sus scrofa domestica*) are provided herein: Amide I/PO_4_ = 0.168 ± 0.000; IRSF = 3.235 ± 0.021; C/P = 0.231 ± 0.001; C/C = 0.736 ± 0.026; FWHM(*ν*_3_PO_4_) = 114.80 ± 2.42; HPO_4_1118 = 0.023 ± 0.000; HPO_4_1145 = 0; HCP = 0.098 ± 0.002; PCP = 0.073 ± 0.002; Cell volume = 532.5; a-axis = 9.437; c-axis = 6.904.

### Classification based on machine learning algorithms

Machine Learning (ML) models are used to identify patterns and relationships in data with the goal of making predictions or classifications (among other tasks) based on new information. A model is the result of training a dataset using an algorithm, which is the process or set of steps that adjusts the model’s parameters. While the algorithm defines how learning occurs from the data, the model encapsulates the learned knowledge and is used to perform specific tasks. When these methods are used as classification tools, they are very robust. Moreover, with respect to the type and size of samples supervised, ML models encounter fewer issues than other traditional statistical methods or unsupervised ML methods. This makes them very useful in addressing archaeological problems^44^.

The application of ML to biostratinomic problems has already been addressed in several studies on surface modifications, bone breakage, and skeletal profiles^44–47^. Similarly, they have been applied to diagenetic problems in both archaeology and forensics^13,48,49^, demonstrating important results in the field of classification and prediction.

In this study, we used 9 algorithms that have been identified as the most powerful classifiers available, allowing us to compare the variability of results and samples: NNET (neural networks), SVM (support vector machines), KNN (k-nearest neighbour), RF (random forest), DTC5. 0 (decision trees using the C5.0 algorithm), LDA (linear discriminant analysis), MDA (mixture discriminant analysis), PLS (partial least squares), NB (naive Bayes). Due to the differences in the way each algorithm processes the data set, different taphonomic aspects can be understood. Therefore, it is necessary to consider those algorithms that give inferior results when interpreting an assemblage. The training of all these classification models was performed using the “caret” library in R language^50^.

The operation was based on dividing the set of samples into 70% to be used for training and the remaining 30% for testing. For this purpose, the data are first centred and scaled. The accuracy value obtained checks the precision of the classification models, a value between 0 and 1. The closer to 1, the closer to a 100% correct classification of the samples. The kappa value is an index that reflects the possibility of a prediction occurring only by chance. This value ranges from − 1 to 1, where 1 indicates perfect classification, − 1 represents a completely inverted model that systematically misclassifies all labels, and 0 corresponds to a classification that is entirely random. Cross-validation has been used with ten folds and 10 repetitions to improve the quality of the analyses, overcoming the model’s overfitting. In order to create accurate models, we used the function “tuneLength” from the ‘caret’ library 45 to create 10 different random models with different hyperparameter configurations of all the algorithms (except LDA, as it does not allow the configuration of the hyperparameters to be changed), which can be compared through kappa agreement index. In addition, the values of sensitivity (true positive rate), specificity (true negative rate), and balanced accuracy (precision of each category analysed) are considered. Note that the final configuration of the best-fit model (i.e. the selected hyperparameters) has been included in the supplementary files for all algorithms, along with the complete script used in this study (Table S3).

## Results

**Fig. 3:**
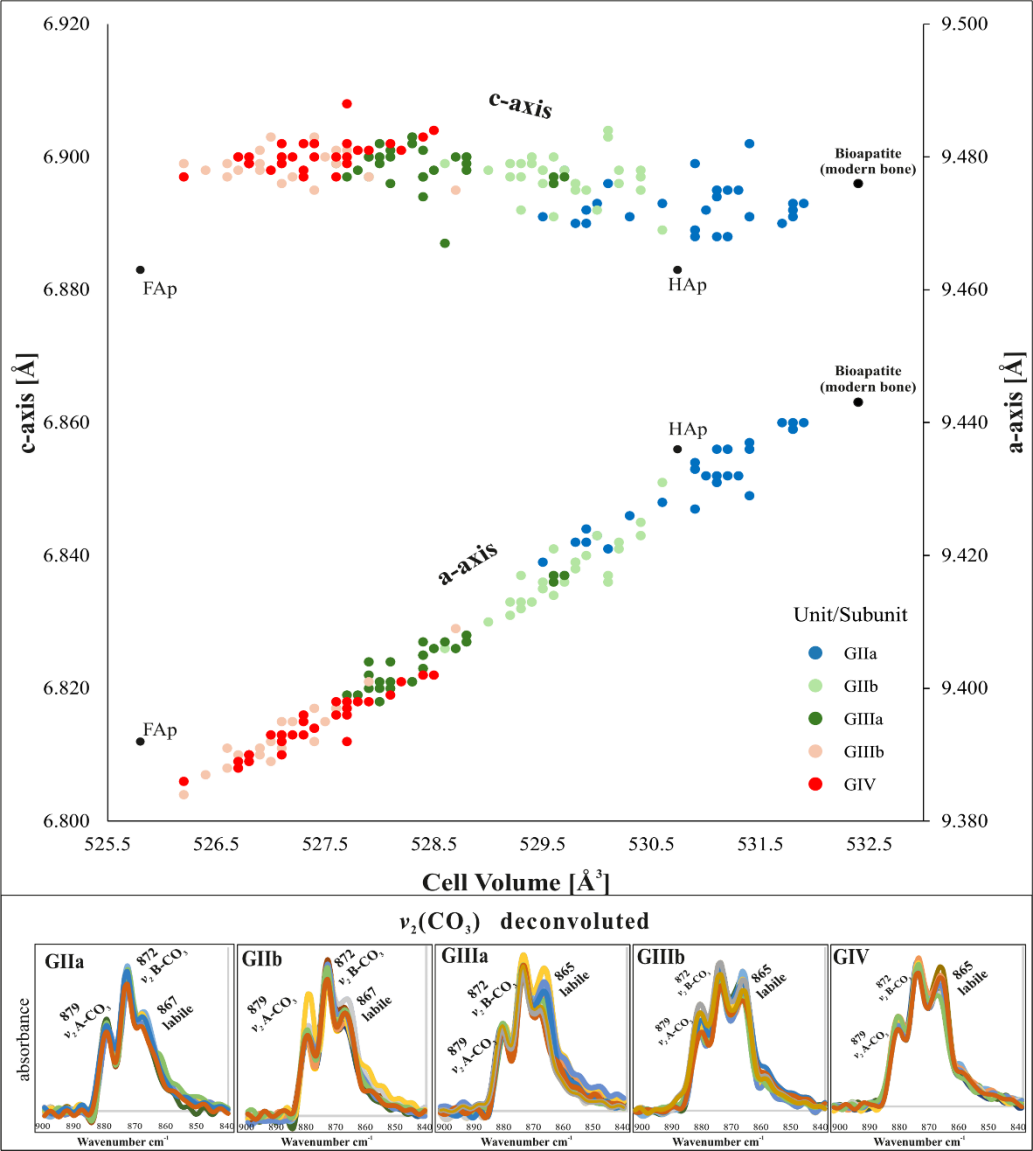
>Dependence of the apatite crystallographic a-axis and c-axis values on the unit cell volume of fossil bones from the Galería site’s stratigraphical sequence. An adult pig femur (*Sus scrofa domestica*) is used as a modern bone reference (Bioapatite). FAp (Fluorapatite reference), HAp (hydroxylapatite reference). Refer to Fig. S2 to view the diffractograms. Below, the deconvolution of the _*v*2_(CO_3_) region in the FTIR spectrum for each unit is shown. 879 cm^−1^ refers to type-A carbonates, 872 cm^−1^ to type-B carbonate substitution. Note that the carbonate band at 865 cm^−1^ is more defined in the bones bearing F^−^ (GIII and GIV units).

**Fig. 4:**
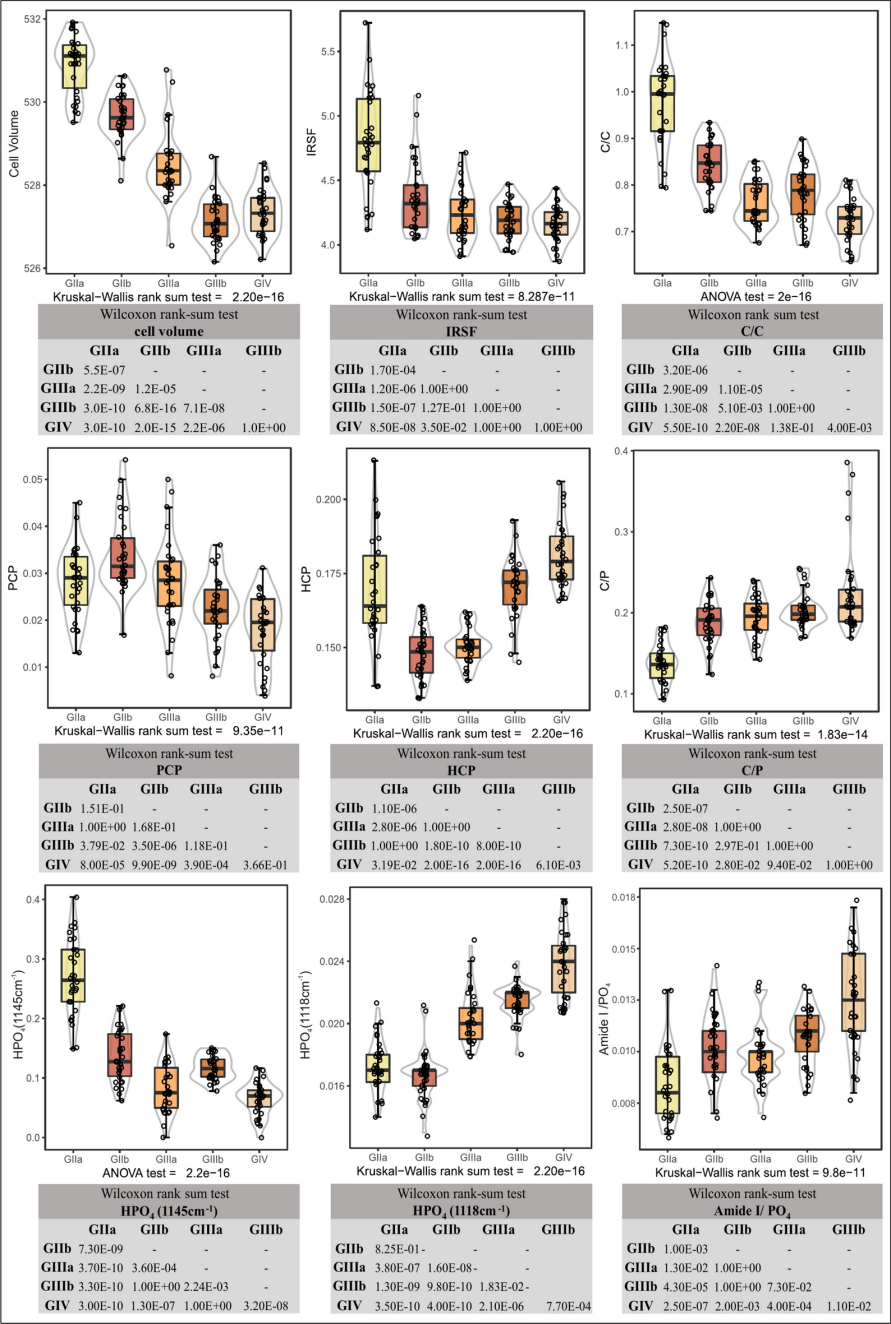
Box plot of diagenetic parameters determined from fossil bone and pairwise comparison between stratigraphical sequence. The rest of the parameters are shown in Fig. S1 and Table S2.

The main chemometric and mineralogical results of the analysis of fossil bone samples obtained by FTIR and XRD are summarized in Table 2, Figs. 4, S1, S2, and Table S2. The infrared spectra primarily showed bands attributed to phosphate and carbonate groups, with minor contributions from amides. Specifically, peaks associated with carbonates were observed at 1450 cm^−1^(*v*_3_CO_3_), 1415 cm^−1^(*v*_3_CO_3_), 872–878 cm^−1^(*v*_2_CO_3_) and 712 cm^−1^(*v*_4_CO_3_), while peaks at 1030 cm^−1^(*v*_3_PO_4_), 960 cm^−1^(*v*_3_PO_4_), 604 cm^−1^ and 565 cm^−1^ are attributable to phosphate bands (*v*_4_PO_4_). Finally, peaks at 1118 cm^−1^ and 1145 cm^−1^, observed in the deconvoluted spectra, are attributed to *v*_3_(PO_4_) bands associated with HPO_4_ (Fig. S3). The ~ 1640 cm^−1^ peak is attributable to Amide I and the 630 cm^−1^ peak is associated with hydroxyl groups (exclusively in unit GII). Furthermore, using the 2nd derivative analysis of the *v*_4_PO_4_ region, after Savitzky-Golay smoothing, allowed us to confirm that bone mineral was mainly carbonate hydroxylapatite for unit GII, while samples from GIIIa onwards presented F-containing phases^31,42,51–53^ (Figs. 2 and 3). Higher intensities of these bands can be observed in units GIII and GIV compared to GII, suggesting a mix of crystalline phases in the bone assemblages of units GIII and GIV (Fig. 2). Similarly, the apatite unit cell parameters (determined by XRD; Table S1; Fig. 3) demonstrate a decrease in the unit cell volume in relation to the increased incorporation of F^−^ in the apatite crystal lattice within the bone assemblage of each subunit. The increase of F^−^ in the bone mineral remains occurs towards the top of the stratigraphic sequence. Fossil bones found in stratigraphic units GIIIb and GIV have a higher mixed phase compared to subunits GIIa and GIIb, which have values closer to hydroxylapatite than fluorapatite. The ranges and averages for each unit are expressed in Fig. 4 and Table 2. A progressive decrease in a-axis and unit cell volume values is observed as the stratigraphic sequence increases until GIIIb is reached. The results of the Wilcoxon sum test for apatite unit cell volume and a-axis show differences between all stratigraphic units (see Fig. 4 and Table S2) except GIIIb and GIV.

**Table 2:** Summary of fossil bone property values (mean ± SD) for each unit/subunit.

	GIIa	GIIb	GIIIa	GIIIb	GIV
IRSF	4.803 ± 0.386	4.362 ± 0.281	4.242 ± 0.209	4.186 ± 0.144	4.161 ± 0.134
C/P	0.138 ± 0.024	0.188 ± 0.027	0.194 ± 0.026	0.204 ± 0.022	0.226 ± 0.056
Calcite/PO_4_	–	0.002 ± 0.005	0.003 ± 0.007	0.003 ± 0.008	0.013 ± 0.002
OH/PO_4_	0.125 ± 0.078	0.019 ± 0.051	–	–	–
HPO_4_1118	0.017 ± 0.002	0.017 ± 0.002	0.020 ± 0.002	0.021 ± 0.001	0.024 ± 0.002
HPO_4_1145	0.270 ± 0.065	0.136 ± 0.043	0.082 ± 0.040	0.117 ± 0.020	0.066 ± 0.028
C/C	0.977 ± 0.093	0.843 ± 0.052	0.764 ± 0.048	0.785 ± 0.060	0.725 ± 0.048
PCP	0.028 ± 0.007	0.034 ± 0.008	0.028 ± 0.009	0.022 ± 0.007	0.018 ± 0.007
HCP	0.170 ± 0.018	0.148 ± 0.009	0.151 ± 0.006	0.170 ± 0.010	0.181 ± 0.011
FWHM(*ν*_3_PO_4_)	69.437 ± 4.111	72.962 ± 2.889	70.788 ± 4.052	68.850 ± 2.719	66.318 ± 2.768
Amide I/PO_4_	0.009 ± 0.002	0.010 ± 0.002	0.010 ± 0.001	0.011 ± 0.001	0.013 ± 0.002
Cell volume (A^3^)	530.9 ± 0.7	529.7 ± 0.5	528.5 ± 0.9	527.1 ± 0.5	527.4 ± 0.6
a-axis (Å)	9.431 ± 0.007	9.417 ± 0.006	9.405 ± 0.008	9.393 ± 0.005	9.394 ± 0.004
c-axis (Å)	6.892 ± 0.003	6.897 ± 0.003	6.899 ± 0.003	6.899 ± 0.002	6.900 ± 0.002

The value units are dimensionless in the case of ratios and area calculations in FTIR, except for FWHM(ν_3_PO_4_), which is expressed in cm^−1^.

Regarding the infrared data, the results of the several indices used for crystallinity and phosphate phase (IRSF, HCP, PCP, HPO_4_, FWHM(*ν*_3_PO_4_)) showed significant differences across all fossil bone assemblages (Fig. 4 and Table 2). Although the results suggested a tendency for apatite crystallinity (IRSF) to increase as the remains get older in the stratigraphic sequence, the Wilcoxon test showed that only the GII unit exhibits dissimilarities in contrast to the other units. On the other hand, GIIIa, GIIIb, and GIV demonstrate no differences through the IRSF (Fig. 4). Statistically significant differences (Kruskal–Wallis = 2.20E−16) were found through highly crystalline phosphates (HCP), excluding the comparison between GIIb-GIIIa (Wilcoxon test = 1) and GIIa and GIIIb (Wilcoxon test = 1). The variations in poorly crystalline phosphates (PCP) are less noticeable (Kruskal–Wallis = 9.35E−11). However, statistically significant differences were found among the subunits GIIa-GIIb (Wilcoxon test = 1.10E−06), GIIIa-GIIIb (Wilcoxon test = 0.000), as well as GIIIb-GIV (Wilcoxon test = 0.006). The FWHM(*ν*_3_PO_4_), which considers the width of the phosphate band at ~ 1010 cm^−1^, is related to the order/disorder of the crystalline lattice^28^, with a value of approximately ~ 115 cm^−1^ in modern bones. The results indicated that the GIIIb and GIV units differ from the others, and there are no statistically significant differences between GIIa-GIIIa-GIIIb (Wilcoxon test = 1), and GIIIa-GIIIb (Wilcoxon test = 0.496).

**Fig. 5:**
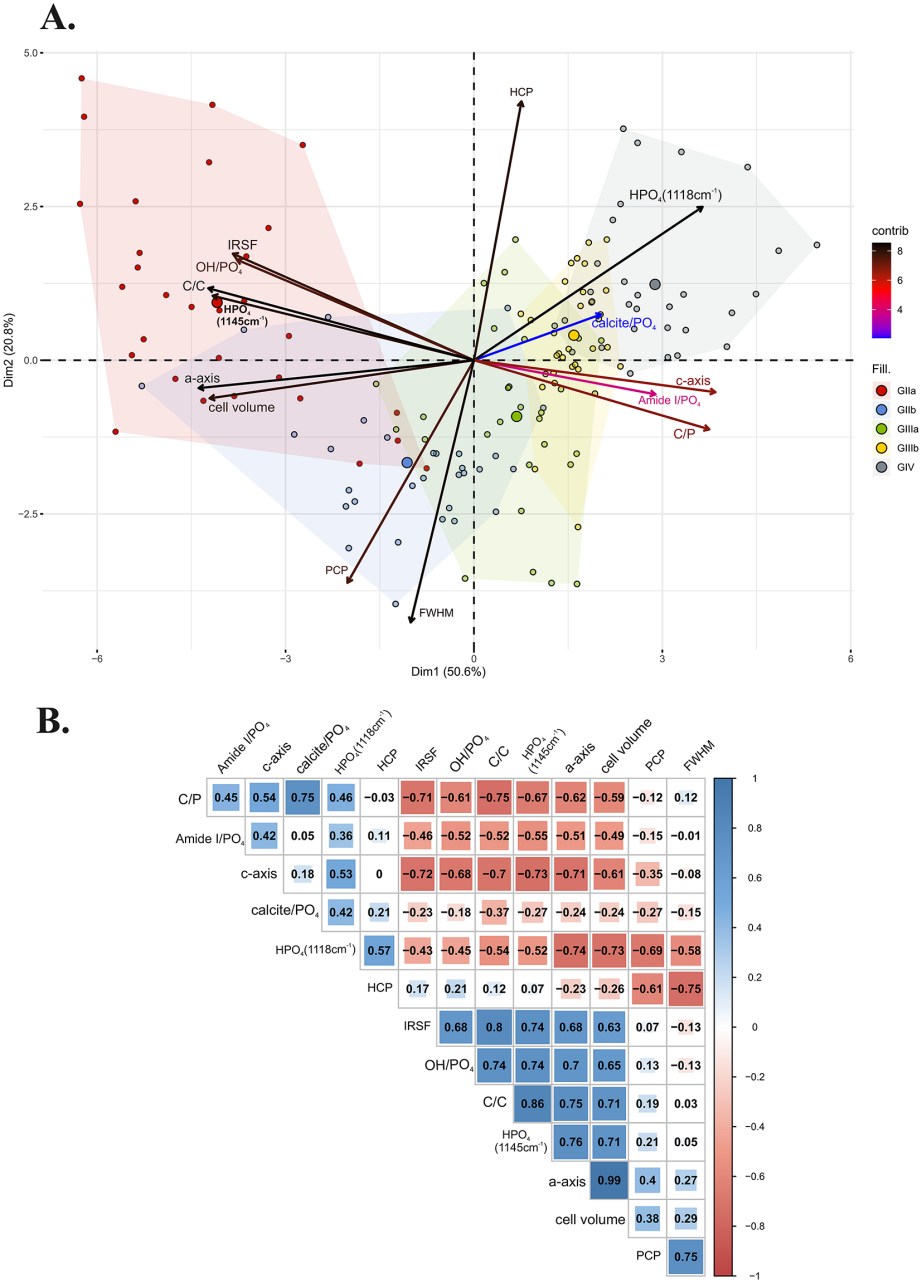
(**A**) Principal component analysis (PCA) showing the dispersion of the bone samples according to all the diagenetic related chemometric parameters used in this study. The darker a vector is, the more it contributes to the spatial distribution. (**B**) Correlation matrix for all parameters used.

Finally, acid phosphate, also known as monohydrogen phosphate (HPO_4_) in bone mineral is a factor to consider in bone diagenesis, as the substitution of phosphate groups with HPO_4_ induces distortions in the apatite crystal lattice^54,55^. Two positions for acid phosphate ions have been recorded around 1118 cm^−1^ and 1145 cm^−1^ for the *v*_3_PO_4_ domain^35,36,56^. The HPO_4_ content around 1118 cm^−1^ is correlated with the a-axis parameter (r^2^ = − 0.74) and the signal of poorly crystalline phosphates (r^2^ = 0.69) (Fig. 5). The HPO_4_ ions at 1145 cm^−1^ are correlated with the substitution of type-A and type-B carbonates (r^2^ = 0.86) and with the a-axis parameter (r^2^ = 0.76). The HPO_4_ content showed statistically significant differences for all sets. The case of HPO_4_ (1118 cm^−1^) allowed for the differentiation of all the sets except GIIa-GIIb (Wilcoxon test = 8.25E−01). Furthermore, the case of HPO_4_ (1145 cm^−1^) allowed for the differentiation of all the sets except GIIIa—GIV (Wilcoxon test = 1).

Regarding carbonate content in bone mineral, the indices carbonate to phosphate (C/P) and the A + B carbonates ratio (C/C) have enabled the differentiation of assemblages and the observation of distinct dynamics among geological units (Fig. 4). The bone assemblage of the GIIa subunit exhibits a loss of carbonate (C/P 0.14 ± 0.02). Starting from the GIIb subunit, the content becomes more stable (C/P 0.19 ± 0.03) and only allows for differentiation between GIIb and GIV (Wilcoxon test = 0.028). The C/C index, which relates type-A carbonates (substitution of OH- groups) and B-type carbonates (substitution of PO_4_^3−^), has revealed differences among all bone assemblages (Welch’s ANOVA test = 2e−16) (Fig. 4). The C/C ratio decreases as the stratigraphic sequence progresses (Table 2), with a higher content of A-type carbonates found in the bone remains of the GIIa subunit. Additionally, there is a band development around 865 cm^−1^ in the fossil bone remains of the GIII and GIV units (Fig. 3).

Calcite, as a separate mineral phase, is detected through the vibrational mode *v*_4_CO_3_ at 712 cm^−1^. The results indicate a high content of calcite in the bone assemblage of the GIV unit (ranging from 0 to 0.073 with an average of 0.013 ± 0.021), as compared to the bone assemblages of GIIIa (ranging from 0 to 0.022 with an average of 0.003 ± 0.007), GIIIb (ranging from 0 to 0.032 with an average of 0.004 ± 0.002), GIIb (ranging from 0 to 0.019 with an average of 0.002 ± 0.001), and the absence of calcite in the remains of GIIa (Fig. S1).

Finally, OH^−^ libration is common in the fossil bone remains of the GIIa subunit (23 out of 30 with an average of 0.13 ± 0.08) and some in the GIIb subunit (4 out of 30 with an average of 0.02 ± 0.05). This however does not occur in the rest of the sequence. This index is correlated with C/C (r^2^ = 0.74), HPO_4_(1145 cm^−1^) (r^2^ = 0.74), and the a-axis (r^2^ = 0.71), which may indicate an arrangement of carbonate ions and acid phosphate ions along the a-axis, especially in the remains of the GIIa subunit.

Concerning the organic matter contents, the Amide I/PO_4_ ratio falls below 0.02 ± 0.00, indicating minimal organic matter preservation. Nevertheless, the findings demonstrate statistically significant distinctions in the Amide I amount between units GIV and GIIa (Wilcoxon test = 2.50E−07). Conversely, no statistically significant differences were observed among subunits GIIb, GIIIa, and GIIIb. Notably, it is within unit GIV that fossil bones have retained a higher Amide I content (0.013 ± 0.002) (Fig. 4).

### Principal components analysis results

The PCA explained 71.4% of the variance with two principal components, revealing the distribution of samples around two dynamics (Fig. 5a, Table S2). On one side, subunits GIIa and GIIb are positioned on the left half of the plot, associated with indices indicating more defined chemical and structural changes of bone mineral compared to units GIII and GIV (IRSF, C/C, HPO_4_-1145 cm^−1^, a-axis, and unit cell volume contributed to their distribution). On the other side, subunits GIIIa, GIIIb, and unit GIV are positioned on the right half of the plot, linked primarily to carbonate content indices C/P, HPO_4_-1118 cm^−1^, and HCP. This illustrates two trajectories within this stratigraphic sequence: one related to hydroxylapatite precipitation and carbonate dissolution, and another one related to the absorption of exogenous carbonates and fluoride ions. The means of each group are distributed differently and the means of GIIIb and GIV are closer to each other. The variables contributing to the distinct distribution of GIIb compared to GIIa and GIIIa compared to GIIIb are FWHM(*ν*_3_PO_4_) and PCP, indices related to the crystallinity of bone mineral.

### Machine learning classification results

All the results of the ML analysis are presented in supplementary files (Table S3). The classification analyses of bone samples using ML algorithms yielded consistent results across the different methods (Table 3). As demonstrated in the PCA and the correlation matrix of variables, a strong relationship (≥ 0.75) exists among different variables. Furthermore, several variables express the same information in the component space (Fig. 5). Therefore, there is multicollinearity in the results that may lead to data overfitting^57^. Taking this into account, we removed variables with a correlation ≥ 0.75, either positive or negative, those that contribute little to the component space, and exclusive variables, i.e., those not present in all fossil bone assemblages (Amide I/ PO_4_, IRSF, C/P, calcite/PO_4_; OH^−^ libration, C/C, FWHM(*ν*_3_PO_4_), c-axis, a-axis). Thus, the models considered the variables cell volume, HCP, PCP, HPO_4_ (1118 cm^−1^) and HPO_4_ (1145 cm^−1^). The least accurate results (Tables 3 and S3) were obtained with the PLS algorithm (accuracy = 0.69; kappa = 0.61) and DTC5.0 (accuracy = 0.76; kappa = 0.69). In contrast, the models that best classified the assemblages were NNET (accuracy = 0.89; kappa = 0.86) and MDA (accuracy = 0.87; kappa = 0.83). The remaining algorithms yielded similar results: SVM (accuracy = 0.84; kappa = 0.81), NB (accuracy = 0.84; kappa = 0.81), RF and LDA (accuracy = 0.82; kappa = 0.78), and KNN (accuracy = 0.80; kappa = 0.75). In other words, neural networks and the mixture discriminant analysis models were better classifiers.

**Table 3:** Accuracy and kappa values provided by the ML algorithms used to classify the different units from Galería site.

	NNET	SVM	KNN	RF	MDA	NB	LDA	PLS	DTC5.0
Accuracy	0.89	0.84	0.80	0.82	0.87	0.84	0.82	0.69	0.76
Kappa	0.86	0.81	0.75	0.78	0.83	0.81	0.78	0.61	0.69
Accuracy lower	0.76	0.71	0.65	0.68	0.73	0.71	0.68	0.53	0.61
Accuracy upper	0.96	0.94	0.90	0.92	0.95	0.94	0.92	0.82	0.87

Therefore, the most important variables for classification are therefore related to the behaviour of phosphates with the apatite crystal lattice parameters. In both the NNET and MDA models, the importance of variables lies between the apatite unit cell volume and HPO_4_ ions. The performance of each algorithm is presented in Table S3, which includes sensitivity, specificity, precision, and balanced accuracy values in relation to the results for each unit and subunit. It is observed that the difficulty in classifying the fossil bone assemblages lies in distinguishing between the remains of GIIIb and GIV, a result that is also evident through the Wilcoxon rank-sum test (Fig. 4; Table S2). The main differences observed between GIIIb and GIV lie in the arrangement of HPO_4_ ions.

## Discussion

**Fig. 6:**
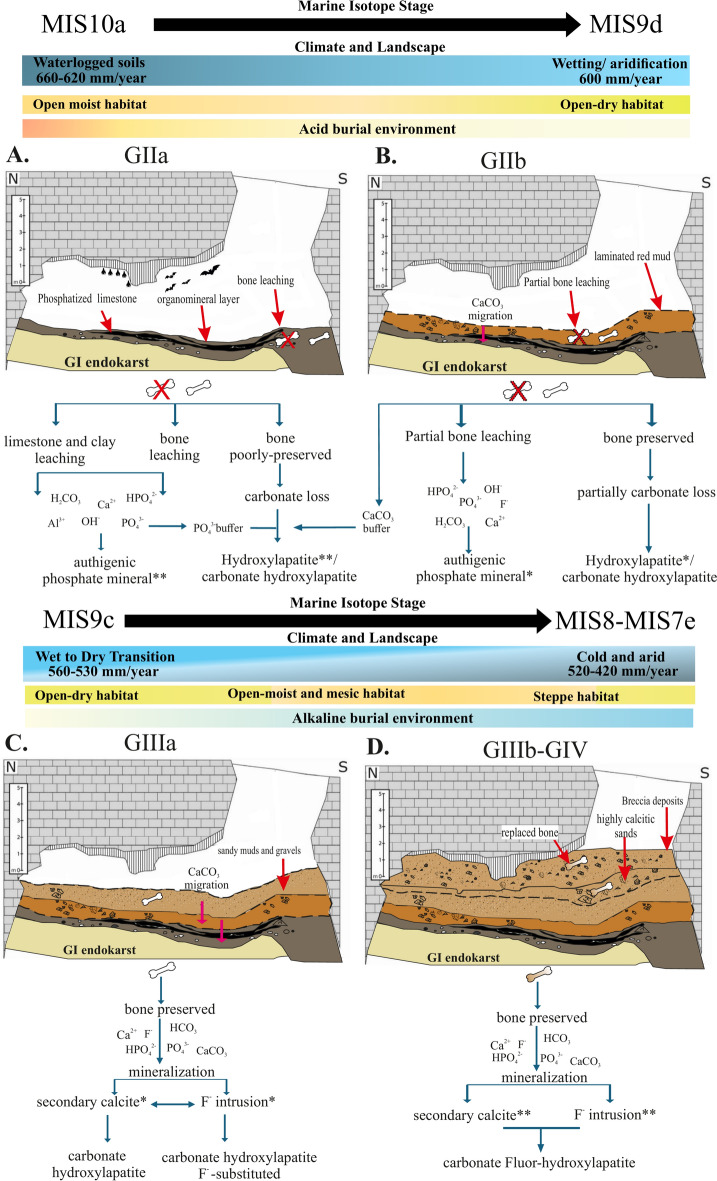
Conceptual model by unit/subunit with climatic and environmental evolution over time. * = moderate process; ** = intense process. Schematic section modified from^60^. (**A**) *GIIa subunit*: Under this acidic environment with organomineral layers, dissolution–precipitation processes lead to the formation of authigenic phosphate minerals and the loss of carbonate from the bone remains, bringing the mineral phase closer to hydroxylapatite. (**B**) *GIIb subunit*: As calcite precipitates, the environment becomes more alkaline, which helps to mitigate the leaching of bone material. (**C**) *GIIIa subunit*: Under increasingly alkaline conditions, calcite precipitation occurs, along with a replacement by F-containing apatite phases. (**D**) *GIIIb-GIV units*: The intensity of fluorine incorporation, along with calcite precipitation, increases. Enhanced calcite precipitation further promotes greater fluorine incorporation.

A taphosystem consists of a preserved association and its external environment^10^. In the case of bone diagenesis in karstic taphosystems, bones are modified by processes stemming from the composition of sedimentary fill, pH, Eh, environmental conditions surrounding caves, soil hydrology, cave hydrological regime, taphon interactions, and the prior taphonomic history before fossil diagenesis^58,59^. Consequently, common features in the preservation mode of remains in karstic taphosystems may be evident, indicating a diagenetic pathway. Furthermore, differences in the preservation mode of remains can occur along a stratigraphic sequence due to the variability of these mentioned characteristics throughout the site’s formation. In the case of the Galería site, the obtained results suggest that the diagenetic history differs for each unit (GII, GIII, and GIV). Additionally, differences have been observed between subunits (GIIa-GIIb, GIIIa-GIIIb). These differences arise during the formation of the stratigraphic sequence, resulting in a diagenetic history that will be discussed for each unit and subunit (Fig. 6).

### From bone mineral to hydroxylapatite in GII unit

The GIIa subunit, framed in MIS 9–10 with a weighted mean age of 313 ± 14 ka by ESR/US method^27^ (combined electron spin resonance and U-series dating), is lithologically defined by layered mudstone beds. This is a mollisol, a soil rich in organic matter with a high water-holding capacity, where acid leaching leads to the alteration of limestone clasts^22,61,62^. The fine-grained sediments, enriched with organic matter, exhibit significant post-depositional alteration, with evidence of in situ leaching, formation of humate microfacies, high biological activity, rhizolites, and authigenic phosphate minerals^23,62^.

Considering the sediment composition, crandallite (CaAl_3_(PO_4_)_2_(OH)_5_·H_2_O), an authigenic mineral formed from phosphate-rich solutions, is also present^22^, indicating a shift in pH from alkaline or near-neutral to acidic^63–65^. Regarding the paleoenvironment and climatic context of this subunit, Bógalo et al.^61^ were able to interpret that the hydrological regime corresponds to 620–660 mm/year. Meanwhile, other studies have observed that it represents humid conditions with an open habitat and waterlogged soils inside the cave^66^. The GIIb subunit, framed in MIS9 and dating back to 237–269 ka by ESR-U-series^27^, presents a milder hydrological regime of around 600 mm/year with periods of wetting and aridification^61^. Similarly, Vallverdú^62^ aligns with the alternation of wet and dry periods for this subunit, defined as vertisol and inceptisol, where a lower content of organic matter is recorded. Thus, in the GII unit, an acidic environment develops, which prevents carbonation (acid leaching) and affects the limestone clasts, altering them. There are processes from which the bone remains are not exempt. Pérez-González et al.^22^ and Vallverdú^62^, pointed out that in the GIIa subunit, there is an absence of carbonates (2%), and as the stratigraphic sequence advances toward GIIb, there is a more pronounced presence (18–32%). Similarly, this process affects the bones, significantly dissolving their carbonate portion in the GIIa subunit and more mildly in the GIIb subunit (Fig. 6a,b), where some fossil bone remains even contain calcite. Thus, it is observed that the preserved remains in GIIa have undergone structural carbonate loss (Fig. 4), while the dissolved remains have contributed to the formation of authigenic phosphate minerals. The faunal remains also suggest significant bone dissolution and loss. Out of the 2704 bone remains recovered from the GII unit, only 2.7% belong to subunit GIIa^67^. In subunit GIIb, there is a proportion of 83.4% (n = 2632) of bone remains compared to 16.6% (n = 523) of lithic industry, a trend that persists throughout the sequence. However, in the GIIa subunit, 19.2% (n = 72) of bone remains were found compared to 80.8% (n = 303) of lithic industry^67^. While the difference between subunits in the lithic assemblage is not particularly large, there is a difference in the proportion of bone remains. Clearly, significant acidic leaching processes have occurred in GIIa, resulting in the loss of bone material in a subunit where much anthropic activity would be expected due to the full functioning of the natural trap as lithic material appears^67–71^.

As the burial history continues, the environmental conditions in GIIb become increasingly dry, alternating between periods of wetting and aridification^61^. Additionally, the diagenesis affecting the bone assemblage in GIIb is less pronounced, with reduced leaching compared to GIIa. Sedimentologically, the GIIb subunit is characterized by layers of red clay, where the limestones exhibiting less alteration and reduced dissolution compared to those in GIIa (Fig. 6a,b). Our results indicate that the deterioration of the carbonate component of bones leads to a precipitation of hydroxylapatite phase. This dissolution-(re) precipitation process is more pronounced in GIIa than in GIIb (as evident from the values of cell volume, c-axis, HCP, IRSF, C/P in Figs. 3 and 4). Even for the fossil bone remains in the GIIa subunit, slight OH^−^ groups libration is detected, which show an advanced deterioration of the fossil bone remains ^72^. The loss of carbonate in the apatite crystal structure results in a pure hydroxylapatite phase, and a shoulder at 630 cm^−1^ can be obtained^73^. This process may also be related to the incorporation of HPO_4_ ions and pH changes^74^. Our findings regarding bone preservation suggest a trajectory characterized by acid leaching/dissolution, decarbonation, and reprecipitation into a more stable apatite phase in the GII unit. The dissolution and (re)precipitation can lead to the formation of authigenic minerals, which may remain stable during subsequent episodes of environmental changes. These processes have been experimentally documented in the early stages of guano-driven diagenesis^75^. The dissolution of bone material stops, enabling the fossilization of remains once the pH is buffered by the migration of CaCO_3_ from the overlying units (beginning in GIIb, see Fig. 6b). This buffering facilitates a transition from an acidic environment to a mildly acidic environment where hydroxylapatite precipitates along with other authigenic phosphate minerals. In this way, the leaching processes are reduced in GIIb, allowing for better bone preservation and less limestone alteration.

### Fluorine replacement in GIII and GIV units

A notable shift in the burial history and conditions at the deposition site is observed beginning with the GIII unit. From this unit onward, bone diagenesis and fossilization are dominated by the incorporation of fluorine and calcite (see Fig. 6). GIIIa is situated within MIS9, with a chronology of 231 ± 18 ka TT-OSL (thermally transferred optically stimulated luminescence) and 244 ± 16 ka pIR-IR225 (post-infrared infrared stimulated luminescence)^23^, mainly made up of allochthonous sediments, predominantly containing rounded pebbles. From this unit onward, a transition from wet to dry environments is observed, progressing from GIIIa to GIIIb, reflecting cold and arid conditions by the time GIV is reached^24,61,66^. This should reflect differences in the burial environment and, consequently, in the fossilization of the bone assemblages. According to our results, the main differences between these subunits occur in the substitution of carbonates within the apatite lattice and the arrangement of HPO_4_ ions, which appear to be related to the substitution of A-type and B-type carbonates in the apatite structure. Therefore, in the fossil bone assemblage of GIIIb, there is a higher predominance of A-type carbonates compared to the GIIIa assemblage (Figs. 3 and 4). From this lithostratigraphic unit onwards, leaching is not as pronounced as it is in the GII unit, a fact that is also observed in the lack of chemical alteration in the limestones ^62^. Thus, it can be observed that the burial environment is more alkaline than in GII, leading to the precipitation of calcite, which is also reflected in the bone assemblages. Moreover, the information provided by the Savitzky-Golay 2nd derivative algorithm and the results from the dependence of a-axis and cell volume (Figs. 2 and 3) have allowed us to observe an F^−^ substitution in the fossil bone remains of units GIII and GIV. Therefore, the diagenetic pathway is characterized by coupled dissolution-(re) precipitation processes^76,77^, where OH⁻ groups are replaced by F^−^, resulting in the formation of distinct fluorine-containing apatite phases. (Fig. 6c).

Both the GII and GIII units hold key significance for the interpretation of Middle Pleistocene human groups in Europe. This importance stems from the presence of highlighted Acheulean lithic set^78^ as well as accumulated faunal records crucial for understanding subsistence dynamics^68,79^. Therefore, thorough control of fossilization and preservation pathways is essential for aiding in the resolution of zooarchaeological and taphonomic data. It is also essential for the acquisition of paleoproteomics, isotopes, paleogenetics data, or dating methods^80–84^.

Continuing with the GIV unit, framed in MIS 8–7, the sedimentary dynamics observed in the GIII unit persist, although breccia sediments become more predominant towards the end of the sequence. Several proxies have indicated cold and arid conditions for this event^24,61,66,68^. Regarding cave usage, there is no longer evidence of its recurrent utilization by human groups. Diagenetic dynamics, like sedimentation, closely resemble those observed in the GIIIb subunit. However, differences have been identified between the GIIIb and GIV fossil assemblages, in which with GIV exhibits a higher content of highly crystalline phosphates and HPO_4_ at 1118 cm⁻^1^. Nevertheless, the similarity in their fossilization dynamics can be attributed to rapid sedimentation during the latter part of the sequence, as discussed in previous studies^22,85^. If we consider the behaviour of carbonates in the fossil assemblages, we also identify significant contributions. Concerning the *v*_2_CO_3_ region for the GIII and GIV units, it is worth noting that the 865 cm^−1^ band, attributed to an environment characterized by labile amorphous calcium carbonates ^42,86,87^, exhibits greater definition compared to unit GII, where it is positioned at 867 cm^−1^ and is less well-defined (Fig. 3). This result may suggest that labile carbonates are favoured by ionic substitutions of F^−^ and OH^−^, as previously proposed^31^. However, it should be noted that this region is ambiguous, and ionic interactions can be highly complex^88^, making it challenging to discern the contribution of carbonate ions. Furthermore, calcite precipitation within the bone tissue promotes micro-fracturing and increased porosity, facilitating F^−^ replacement^89^, as observed in the GIV unit. Finally, following the formation of the GIV unit, the GV unit completes the cavity’s infilling, enabling the fossilization of bone remains within the described contexts.

### Overview of the burial history

The Galería site reveals different diagenetic processes that evolved in response to changing burial environments along the stratigraphic sequence (Fig. 6). Crystalline phases began to evolve with the loss of organic material during early diagenesis and were refined during late diagenesis through dissolution-(re)precipitation processes.

In the GII unit (MIS 10a–MIS 9d), acidic and wet conditions dominated, particularly in GIIa, where leaching caused significant loss of bone material and led to the formation of authigenic phosphates. The preserved bone remains exhibit a hydroxylapatite phase with dissolved carbonate (Fig. 6a). As conditions progressed into GIIb, drier environments facilitated pH buffering through CaCO_3_ precipitation, which stabilized the carbonate hydroxylapatite phase in the bone mineral (Fig. 6b), improving bone preservation.

In the most recent units, the burial environments of the GIII and GIV units shifted to slightly alkaline conditions. These units are characterized by calcite precipitation and fluorine replacement into the bone apatite. The dissolution-(re) precipitation processes intensified from GIIIa (MIS 9c) to GIIIb, resulting in the formation of fluorine-containing apatite phases (Fig. 6c,d). The GIIIb and GIV units (MIS 8–MIS 7e) share such a closely related diagenetic trajectory that it suggests rapid sedimentation, ultimately contributing to the complete infilling of the cave.

### Machine Learning-based classification of fossil bones across stratigraphic units

Considering the observed burial history, the diagenetic parameters have enabled the establishment of classification models for the bone assemblages along the stratigraphy. These diagenetic differences allowed machine learning algorithms to classify the assemblages with an accuracy of 0.89 and 0.87 using neural networks (NNET) and mixture discriminant analysis (MDA) algorithms, respectively (Tables 3 and S3). The variables that contribute most to this classification are related to the behaviour of phosphates and the unit cell structure. The evaluation metrics demonstrate the effectiveness of the NNET model with high performance in GIIa and GIIIa (balanced accuracy of 1.00 and 0.96, respectively). Phosphate bands (HCP, HPO_4_ at 1145 and 1118 cm⁻^1^) and apatite unit cell volume were key contributors to the classification. Although GIIIb showed lower sensitivity (0.67), the neural network (NNET) model, optimized with a hidden layer size of 5 and a decay value of 0.1, proved robust and efficient, highlighting the relevance of chemical and structural diagenetic changes in distinguishing units.

At the nanoscale, fossil bone assemblages homogenize as they seek stability within burial environments. This homogenization enables the tracing of common preservation trajectories for fossil bone remains^13,43^. This remains valid even when analysing cortical bone from different mammalian taxa, which, as shown in previous studies^48,59,90^ exhibit compositional variability in vivo. In any event, we can observe that the karstic taphosystem developed at the Galería site differentially preserves its fossil assemblage according to the stratigraphy. While it is true that the wide range of some variables may be attributed to spatial variability at different points within the stratigraphic units, this issue can be addressed in future studies. These machine learning models will allow us to address issues of looting and fossil bone decontextualization, at least for sites in karstic systems.

## Conclusion

This study investigates the different preservation states of fossil bone remains in the Galería deposit. XRD and FTIR chemometric parameters were used to characterize the fossil assemblages within the several stratigraphic units of the Galería site. It was observed that these units showed different patterns of fossilization and preservation in the chemistry and mineralogy of the fossil bone remains. This differential preservation is attributed to different burial environment conditions during the deposit formation.

The oldest unit, the GII unit, is characterised by leaching, carbonate loss, and dissolution–precipitation processes, bringing the bone remains closer to the hydroxylapatite phase. This suggests an acidic and wet burial environment where the dissolution of bone remains leads to the formation of authigenic phosphate minerals such as crandallite. The GIII and GIV units are characterised by an incorporation of F^−^ and CO_3_ into the crystal structure. The results suggest a slightly alkaline burial environment with lower moisture than in the lower part of the sequence.

These differences have enabled the development of supervised Machine Learning classification models for fossil bone remains at the Galería site. The ML algorithms (NNET and MDA) identified apatite crystal lattice parameters and phosphate molecules as the most relevant factors for differentiating fossil bones across the stratigraphic sequence. These models provide a valuable tool for recontextualizing fossil remains and understanding diagenetic processes, with potential applications in other karstic systems.

## Supplementary Information

Supplementary Information 1.

Supplementary Tables.

Supplementary Information 2.

## Data Availability

All data and Rcode are presented in the main text and the Supplementary Information.
