# Supplementary material for: Bone diagenesis and stratigraphic implications from Pleistocene karst systems
Source: Sci Rep. 2025 Feb 14;15:5496. doi: 10.1038/s41598-025-88968-4 (PMC11828995; doi:10.1038/s41598-025-88968-4)
Supplement: Supplementary file 3 — Supplementary Information 2. [file 41598_2025_88968_MOESM3_ESM.pdf]

Supplementary Information

Scientific Reports

Bone diagenesis and stratigraphic implications from Pleistocene karst systems

Héctor Del Valle[1,2*,] Alejandro B. Rodríguez-Navarro[3], Abel Moclán[4,5,6], Paula García-Medrano[1,2,7,8], Isabel Cáceres[2,1]

1 Institut Català de Paleoecologia Humana i Evolució Social (IPHES-CERCA), Zona Educacional 4, Campus Sescelades URV (Edifici W3), 43007 Tarragona, Spain

2 Universitat Rovira i Virgili, Departament d'Història i Història de l'Art, Avinguda de Catalunya 35, 43002 Tarragona, Spain

3 Departamento de Mineralogía y Petrología, Universidad de Granada, 18002 Granada, Spain.

4 Laboratoire PALEVOPRIM, Université de Poitiers and CNRS, Poitiers, France

5 Institute of Evolution in Africa (IDEA), University of Alcalá de Henares, Covarrubias 36, 28010, Madrid, Spain

6 Institute of Evolution in Africa (IDEA), University of Alcalá de Henares, Covarrubias 36, 28010, Madrid, Spain

7 UMR 7194 HNHP (MNHN-CNRS-UPVD), Département Homme et Environnement, Museum National d'Histoire Naturelle, Paris, France

8 Dept. Britain, Europe and Prehistory, British Museum, Frank House, 56 Orsman Road N1 5QJ, London, UK

* Corresponding author: hectorvalleblanco@gmail.com; hdelvalle@iphes.cat

SI 1: Introduction

SI 1.1: Galería site

Galería is one of the sites that make up the archaeopalaeontological complex of the Sierra de Atapuerca (Burgos, Spain). Sierra de Atapuerca is located to the north of the northern plateau at an altitude of 1085 masl. It is composed by several cave systems. Specifically, Galería, along with Gran Dolina and Sima del Elefante, is located on the western side of the Sierra within the railway trench. (Fig.1). These sites were exposed due to the construction of the railway trench in the 19th century, although archaeological excavations did not begin until 1976 and systematically until 1982 when excavation began specifically for the Galería site. Since then, excavations have recovered a rich archaeological record from 1.5 my, providing key information for the knowledge of the Lower and Middle Pleistocene populations on the Eurasian continent [1–3].

Galería is approximately 14 m high, 18 m wide, and over 12 m deep and is divided into three sectors. The central area Trinchera Galería (TG), a small cavity called Trinchera Zarpazos (TZ), and the sinkhole called Trinchera Norte (TN). The stratigraphic sequence belonging to the Middle Pleistocene is composed of six lithostratigraphic units named from GI to GVI [4], containing archaeopalaeontological record from GII to GIV units (Fig.1 and 2). This record is made up to date of about 21000 faunal remains and 3000 lithic tools, ascribed to the Acheulian [3,5–8]. In addition to these, two pre-Neanderthals human remains, a mandible (ATA76-T1H) [9] and an adult neurocranial fragment (ATA95-TZ-GIII-K05-29) [10] are worth mentioning. The study of this sequence has contributed to an important debate on the subsistence strategies of human groups in the European Middle Pleistocene. This has allowed deducing that the cavity was used as a natural trap for the consumption of animals falling into the cavity through TN sinkhole [11,12], reflecting a sporadic and non-intensive occupation dynamic. Thus, as the cave filled in, it lost its effectiveness as a natural trap and occupations became less intense [7].

The characteristics of the lithostratigraphic units themselves are the following. GI unit is a deposit composed of thin yellow beds corresponding to the detrital sediments of the endokarst formation, so no archaeopalaeontological record has been recovered. The Matuyama/Brunhes inversion was located in the space represented by an erosional hiatus in this stratigraphic unit [4,13].

From GII unit onwards, a change in sedimentation can be observed, becoming sedimentation of allochthonous origin until its definitive clogging in GV and GVI. Unit GII is subdivided into GIIa and GIIb, which are characterized by an archaeological richness in faunal and lithic remains [14]. Subunit GIIa consists of fine stratified mudstone beds of diverse red, yellow, and black shades with organo-mineral facies and weathered

limestones [15,16]. This unit was dated at 350-363 Ka and 313±14 Ka by ESR/US method [17] and 503±95 Ka by TL [18]). Subunit GIIb dated at 237-269 ka ESR-U-series [17] is made up of one-meter-thick beds of breccia and red mudstone with lamination and massive sediments.

GIII unit is divided into subunits GIIIa and GIIIb. The multiple dating methods applied in this Unit has contributed to an intense debate about chronology and sedimentation rate depending on the dating method TL, ESR, ESR-US, TT-OSL, pIR-IR_{225} and pIR-IR_{290} [8,17–19]. In general terms, this unit has been framed between 460 ka and 220 ka for GIIIa subunit and 300 ka and 250 ka for GIIIb subunit. In general, this unit is mainly made up of red and yellowish sandy beds with fine gravels, pebbles, and cobbles that are stratified with breccia beds [15]. A rich archaeological record has been recorded in this unit with faunal remains, lithic artifacts, and human remains that decreases as we approach the upper part of the unit. Thus, GIIIa and GIIIb are divided due to the differences in the archaeological record and due to the presence of calcitic sands in the upper part.

GIV unit is made up of yellowish-red massive mudstone beds that are stratified with breccia beds and cemented pebbles. In its upper part it ends with stalagmitic layers. This unit has been dated as 255 ± 21 ka SG TT-OSL and 245 ± 15 ka pIR-IR_{225} and 185±26 ka [18,19]. The archaeopalaeontological record has yielded few faunal remains for this last phase when the natural trap became practically useless [20].

Finally, GV and GIV units correspond to the last infilling event and formation of the edaphic level sealing the cavity. The dating of the upper part in the TZ was 135±13 ka [21].

Regarding the environmental conditions, several authors have offered climatic models from different disciplines for the Galería sequence [15,16,20,22]. In general, we can observe a humid and moderate climate with waterlogged soils in the cave for unit GIIa during MIS9/10. Whereas in unit GIIb, alternating periods of wetting/aridification followed by periods of intensified precipitation in an open landscape have been recorded during MIS9e-MIS9d. Unit GIII suggests a temperate climate with open landscapes and woodland patches. This would be a transition from a humid environment to a drier glacial phase already in GIV from MIS9 to MIS8.

Supplementary Information 1: Figures

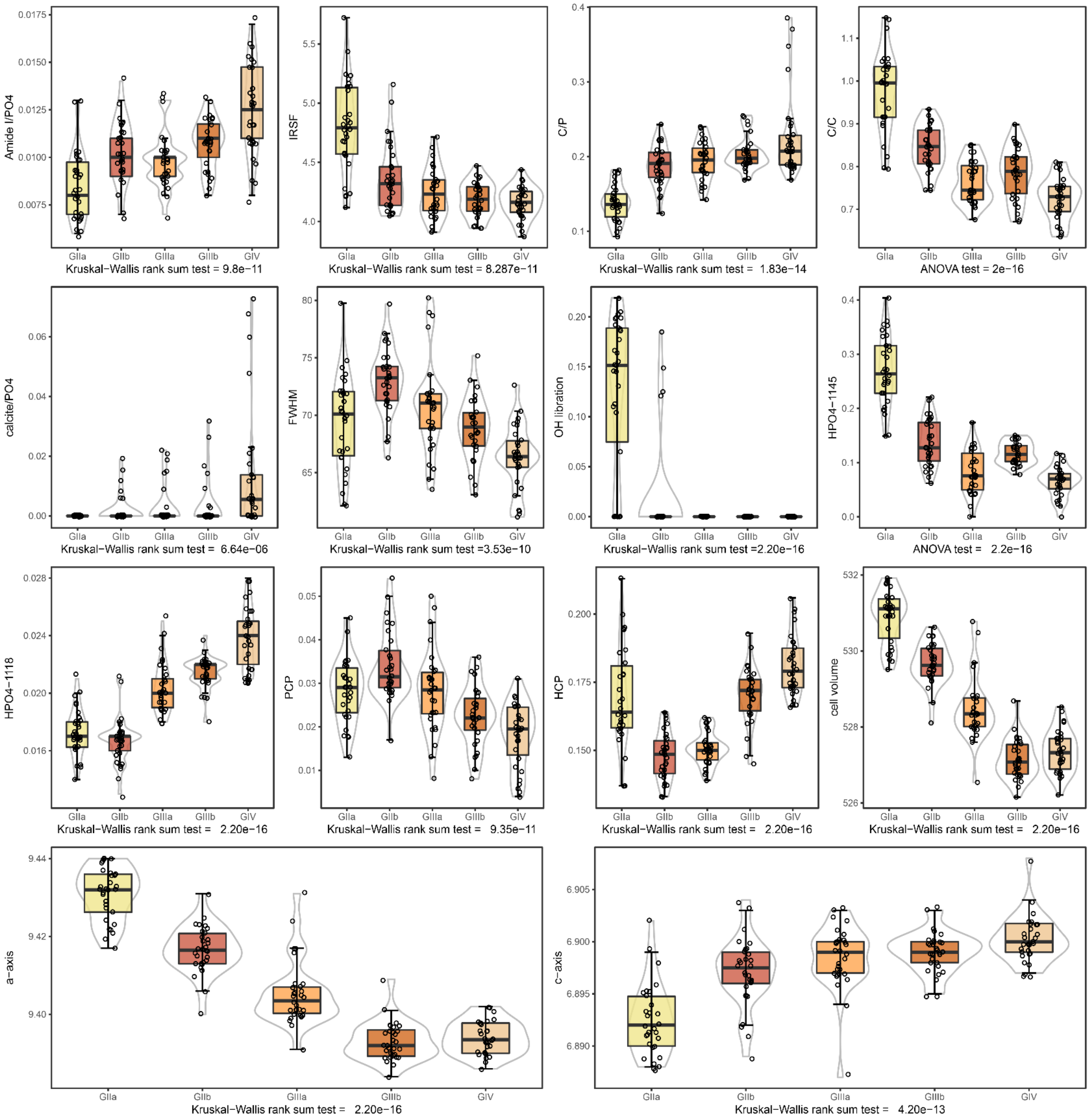

Figure S1. Box plot of all diagenetic parameters used in this study.

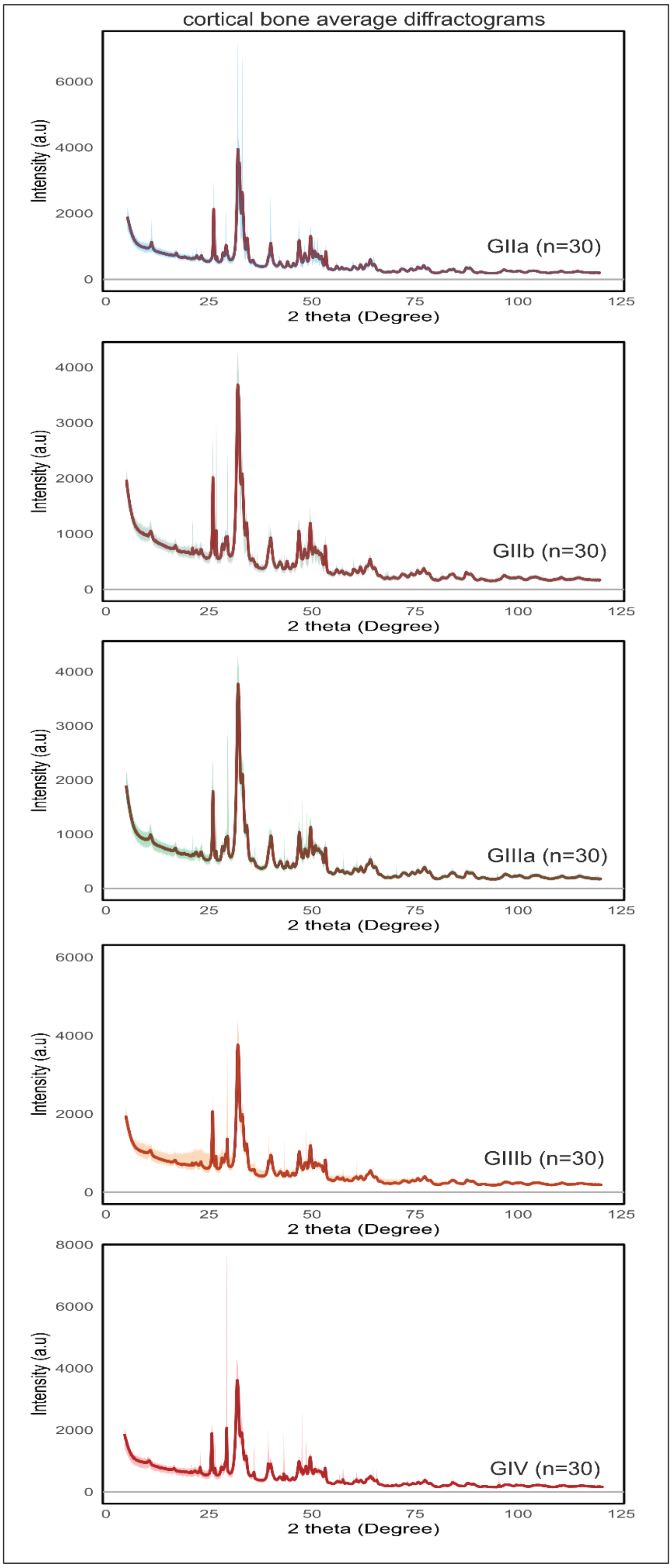

Figure S2. Average bone powder XRD diffractograms from each stratigraphic unit.

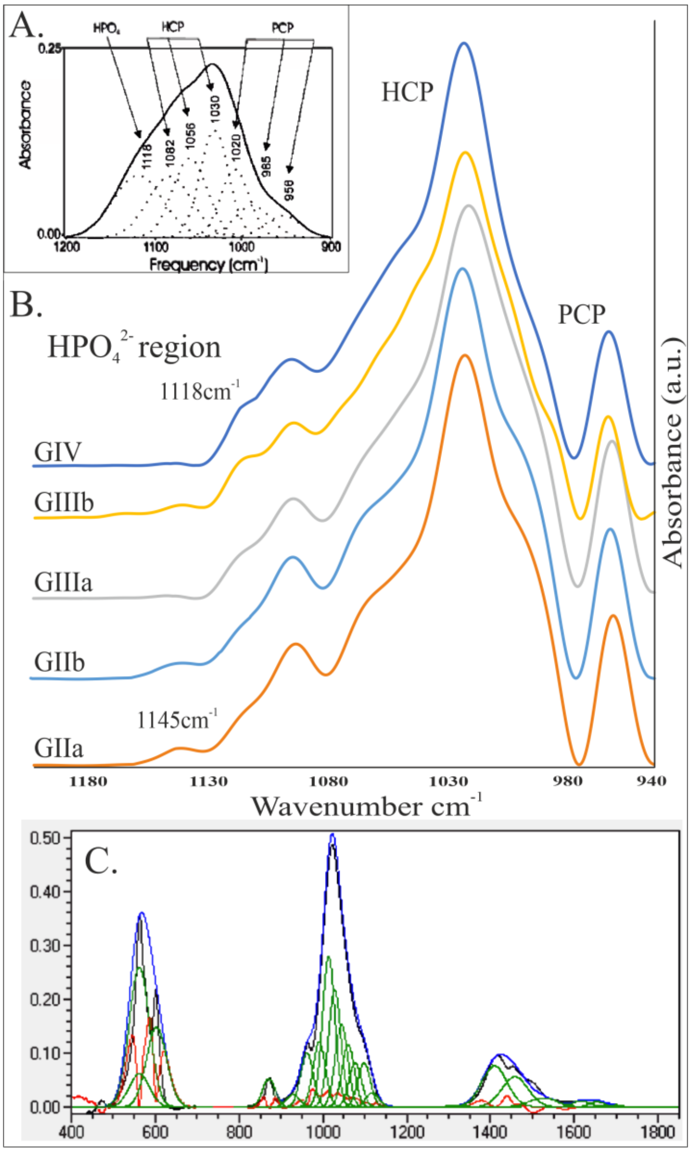

Figure S3. Deconvolution bands of the $_{V3}$PO4 domain. A) The 1200-900 cm^{-1} band region, associated with phosphate groups. HCP and PCP stand for highly and poorly crystalline phosphate, respectively; modified from Rodríguez-Navarro et al.[23]. B) Example of deconvolution bands for each lithostratigraphic unit studied. C) Overlapping peaks resolved and their integrated areas measured using specifically designed curve fitting software.

Supplementary Tables

The supplementary tables with all the data and analyses performed in this work can be found in the Supplementary Tables file. The file contains the following tables:

Table S1. Identification and diagenetic parameters of all the bone samples. This table shows the value for each parameter together with the identification reference and the anatomical and taxonomic identification of each element used.

Table S2. Statistical analysis. This table contains the summary of all the applied analyses. It includes the Shapiro-Wilk normality test, the homogeneity of variance test, the Kruskal-Wallis and Welch's ANOVA tests for each case, the pairwise comparisons using the Wilcoxon rank sum test, and the summary of the PCA and correlation matrix analyses.

Table S3. Machine learning model. This table contains all the results required for each of the 9 ranking algorithms: NNET (neural networks), SVM (support vector machines), KNN (k-nearest neighbors), RF (random forest), MDA (mixture discriminant analysis), NB (naive Bayes), LDA (linear discriminant analysis), PLS (partial least squares), and DTC5.0 (decision trees using the C5.0 algorithm).

References

1. Carbonell, E. *et al.* The first hominin of Europe. *Nature* **452**, 465–469 (2008).

2. Rodríguez, J. *et al.* One million years of cultural evolution in a stable environment at Atapuerca (Burgos, Spain). *Quat. Sci. Rev.* **30**, 1396–1412 (2011).

3. Ollé, A. *et al.* The Early and Middle Pleistocene technological record from Sierra de Atapuerca (Burgos, Spain). *Quat. Int.* **295**, 138–167 (2013).

4. Pérez-González, A. *et al.* Géologie de la Sierra de Atapuerca et stratigraphie des remplissages karstiques de Galería et Dolina (Burgos, Espagne). *Anthropologie* **105**, 27–43 (2001).

5. García-Medrano, P. *et al.* The earliest Acheulean technology at Atapuerca (Burgos, Spain): Oldest levels of the Galería site (GII Unit). *Quat. Int.* **353**, 170–194 (2014).

6. García-Medrano, P., Ollé, A., Mosquera, M., Cáceres, I. & Carbonell, E. The nature of technological changes: The Middle Pleistocene stone tool assemblages from Galería and Gran Dolina-subunit TD10.1 (Atapuerca, Spain). *Quat. Int.* **368**, 92–111 (2015).

7. García-Medrano, P., Cáceres, I., Ollé, A. & Carbonell, E. The occupational pattern of the Galería site (Atapuerca, Spain): A technological perspective. *Quat. Int.* **433**, 363–378 (2017).

8. Ollé, A. *et al.* The European Acheulean from the Atapuerca perspective. Two steps forward, one step back. *Quat. Int.* **411**, 316–328 (2016).

9. Bermúdez de Castro, J. M. & Rosas, A. A human mandibular fragment from the

Atapuerca Trench (Burgos, Spain). *J. Hum. Evol.* **22**, 41–46 (1992).

10. Arsuaga, J. L., Gracia, A., Lorenzo, C., Martínez, I. & Pérez, P. J. Resto craneal humano de Galería/cueva de Zarpazos (Sierra de Atapuerca, Burgos). in *Atapuerca: Ocupaciones Humana y Paleoecología del yacimiento de Galería, vol.7* (eds. Carbonell, E., Rosas, A. & Díez, J. C.) 233–236 (Memorias de la Junta de Castilla y León, 1999).

11. Cáceres, I. Tafonomía de yacimientos antrópicos en karst. Complejo Galería (Sierra de Atapuerca, burgos), Vanguard cave(Gibraltar) y Abric Romaní (Capellades, Barcelona). (Universitat Rovira i Virgili, 2002).

12. Huguet, R. *et al.* Le gisement de Galería (Sierra de Atapuerca, Burgos, Espagne) : Un modèle archéozoologique de gestion du territoire au Pléistocène. *Anthropologie.* **105**, 237–257 (2001).

13. Campaña, I. *et al.* Reconstructing depositional environments through cave interior facies: The case of Galería Complex (Sierra de Atapuerca, Spain). *Geomorphology* **440**, (2023).

14. Cáceres, I. *et al.* El yacimiento de Galería (Sierra de Atapuerca, Burgos, España): un enclave para la obtención de recursos cárnicos en el Pleistoceno Medio. in *1a Reunión de científicos sobre cubiles de hiena (y otros grandes carnívoros) en los yacimientos arqueológicos de la Península Ibérica, Alcalá de Henares,* (vol.13, pp.157-165. Zona Arqueológica, 2010).

15. Vallverdú, J. Soil-stratigraphy in the cave entrance deposits of Middle Pleistocene age at the Trinchera del Ferrocarril sites (Sierra de Atapuerca, Spain). *Quat. Int.* **433**, 199–210 (2017).

16. Bógalo, M. F. *et al.* High-resolution late Middle Pleistocene paleoclimatic record from the Galería Complex, Atapuerca archaeological site, Spain - An environmental magnetic approach. *Quat. Sci. Rev.* **251**, (2021).

17. Falguères, C. *et al.* Combined esr/u-series chronology of acheulian hominid-bearing layers at trinchera galería site, atapuerca, spain. *J. Hum. Evol.* **65**, 168–184 (2013).

18. Berger, G. W. *et al.* Luminescence chronology of cave sediments at the Atapuerca paleoanthropological site, Spain. *J. Hum. Evol.* **55**, 300–311 (2008).

19. Demuro, M. *et al.* New luminescence ages for the Galería Complex archaeological site: Resolving chronological uncertainties on the Acheulean record of the Sierra de Atapuerca, Northern Spain. *PLoS One* **9**, (2014).

20. Núñez-Lahuerta, C., Galán, J., Cuenca-Bescós, G., García-Medrano, P. & Cáceres, I. A bird assemblage across the MIS 9/8 boundary: The Middle Pleistocene of Galería (Atapuerca). *Quat. Sci. Rev.* **293**, (2022).

21. Pérez-González, A. *et al.* Geología y estratigrafía del relleno de Galería de la Sierra de Atapuerca (Burgos). in *Atapuerca: Ocupaciones Humanas y Paleoecología del Yacimiento de Galería, vol.7* (eds. Carbonell, E., Rosas, A. & Díez, J. C.) 31–42 (Memorias de la Junta de Castilla y León, 1999).

22. Expósito, I., Burjachs, F. & Allué, E. Filling in the gaps: The contribution of non-pollen palynomorphs to knowledge about the local environment of the Sierra de

Atapuerca caves during the Pleistocene. *Quat. Int.* **433**, 224–242 (2017).

23. Rodriguez-Navarro, A. B., Romanek, C. S., Alvarez-Lloret, P. & Gaines, K. F. Effect of in ovo exposure to PCBs and Hg on clapper rail bone mineral chemistry from a contaminated salt marsh in coastal Georgia. *Environ. Sci. Technol.* **40**, 4936–4942 (2006).
